# Nuclear Medicine Tracers Targeting Tumor-Associated Macrophages

**DOI:** 10.3390/pharmaceutics18091138

**Published:** 2026-09-10

**Authors:** Dai Shi, Wujian Mao, Yutao Xie, Pan Zhou, Minqiang Hu, Yuxia Liu, Dengfeng Cheng

**Affiliations:** 1 Department of Nuclear Medicine, Shanghai Clinical Research and Trial Center, Shanghai 201203, China; sdshi222@163.com (D.S.); maowujian@foxmail.com (W.M.); xieyt2025@shanghaitech.edu.cn (Y.X.); zhoupan2025@shanghaitech.edu.cn (P.Z.); humq2025@shanghaitech.edu.cn (M.H.); 2 School of Biomedical Engineering & State Key Laboratory of Advanced Medical Materials and Devices, ShanghaiTech University, Shanghai 201210, China; 3 Department of Nuclear Medicine, Zhongshan Hospital Fudan University, Shanghai 200032, China; 4 Department of Applied Chemistry, Shanghai Institute of Applied Physics, Chinese Academy of Sciences, Shanghai 201800, China

**Keywords:** tumor-associated macrophages, nuclear medicine tracer, PET imaging, SPECT imaging

## Abstract

Malignant tumors are the second major disease threatening human health, and their early diagnosis can significantly improve patients’ prognosis. [^18^F]FDG PET/CT is currently the most important method in nuclear medicine for tumor diagnosis and staging. However, due to variations in the glucose uptake characteristics of tumors, some tumors with low FDG uptake (e.g., gastric mucinous adenocarcinoma and indolent lymphoma) are prone to false-negative results. Therefore, new tumor-specific imaging tracers are urgently needed. Due to the significant differences in target expression among various tumor cells, the method of focusing on a single target is unlikely to work for all tumors. Therefore, researchers are exploring the possibility of targeting tumor-associated macrophages (TAMs) in the tumor microenvironment for imaging because this offers several advantages. First, unlike most tumors, which originate from epithelial cells, TAMs arise from monocyte–macrophage polarization, leading to greater target specificity on their surfaces. Second, TAMs are the most abundant non-tumor cells in the tumor microenvironment, ensuring a certain level of expression abundance. Furthermore, numerous preclinical studies and clinical trials targeting TAMs with immunotherapy are currently underway, making the non-invasive assessment of certain TAM targets’ expression levels a critical issue that needs to be addressed. This article summarizes the potential targets and nuclear medicine tracers for targeting TAMs.

## 1. Background

Malignant tumors are still the second largest disease threatening human health, and early diagnosis and treatment are the top priorities. Positron Emission Tomography/Computed Tomography (PET/CT) and Single-Photon Emission Computed Tomography (SPECT/CT) combine anatomic and functional imaging to detect changes in lesion function before anatomic changes occur [1,2]. [^18^F]FDG PET/CT is the most commonly used nuclear medicine examination method in clinics [3]. However, due to the variations in tumors’ glucose uptake characteristics, [^18^F]FDG cannot fully meet clinical needs. For example, some tumors with low [^18^F]FDG uptake are difficult to detect or stage using [^18^F]FDG PET/CT. In addition, [^18^F]FDG PET/CT has limited use in distinguishing inflammation from cancer [4]. To solve this problem, it is necessary to develop new tumor-specific imaging tracers.

In recent years, the tumor immune microenvironment has been the focus of research. TAMs, which account for the largest proportion of immune cells in the tumor microenvironment, have been widely studied [5]. As an important part of innate immunity, macrophages are widely involved in various diseases and can be mainly classified as M1-type macrophages and M2-type macrophages [6]. M1-type macrophages have pro-inflammatory and anti-tumor effects; they secrete a variety of pro-inflammatory cytokines, and phenotypically, they highly express CD86, CD80, etc. [7,8]. M2-type macrophages promote angiogenesis, anti-inflammation, and pro-tumor effects; they secrete anti-inflammatory factors, and phenotypically, they highly express CD206, CCL18, Arginase-1, TREM2, etc. [9,10]. TAMs showed a similar phenotype to M2-type macrophages.

There is a sufficient biological basis for the diagnosis and differential diagnosis of tumors by targeting TAMs. TAMs are differentiated from monocytes, and there are significant differences in biomarkers and expression levels between TAMs and epithelial-derived tumor tissues and normal tissues. In addition, due to the significant differences in macrophage phenotypes between the tumor microenvironment and the inflammatory microenvironment, targeting TAMs is also feasible for the differential diagnosis of tumors and inflammation. A variety of imaging tracers targeting macrophages have been investigated; however, the development of tracers targeting TAMs is in its infancy.

This article summarizes the potential targets and imaging tracers for targeting TAMs. While some of these probes have not been evaluated in tumor animal models, they show promise for future applications and thus are included in this review.

## 2. TREM2

**Figure 1:**
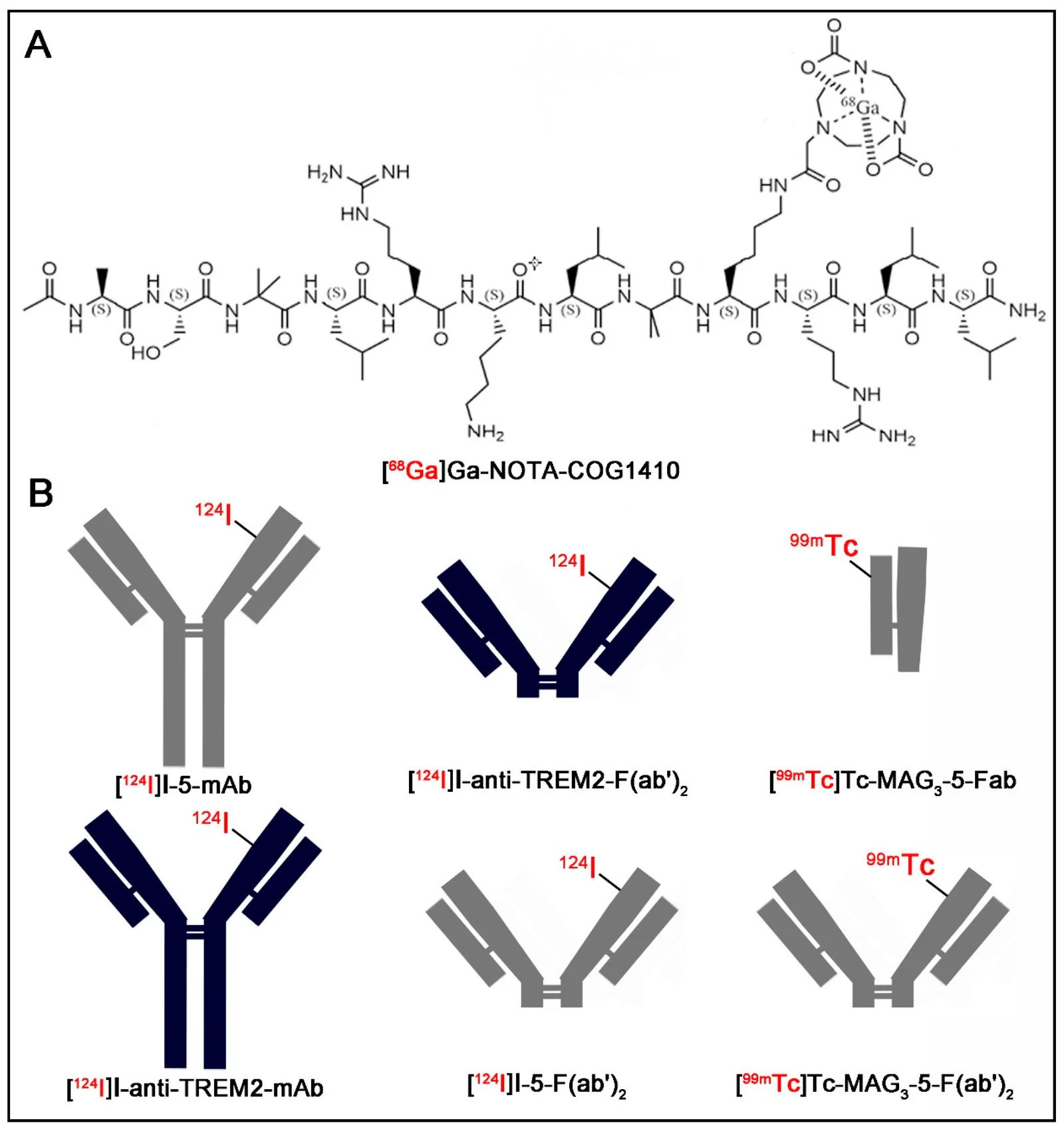
The TREM2-targeting nuclear medicine tracers. The molecular formula of [^68^Ga]Ga-NOTA-COG1410 (**A**). The radioimmunoimaging tracers targeting TREM2 (**B**).

Triggering receptor expressed on myeloid cells-2 (TREM2) belongs to the immunoglobulin superfamily as a one-way transmission membrane receptor with an extracellular domain. In the central nervous system, it was first identified in microglia; in the periphery, it is predominantly expressed on the surface of myeloid cells, including macrophages [11]. Some scholars believe that TREM2 is a surface biomarker of TAMs [11,12]. There is a sufficient theoretical basis for the diagnosis and differential diagnosis of tumors by targeting TREM2. The expression level of TREM2 in most tumor tissues was significantly higher than that in the corresponding normal and inflamed tissues [13]. Therefore, it is theoretically feasible to target TREM2 for the diagnosis of tumors and the differential diagnosis of tumors and inflammation. Shi et al. synthesized the peptide molecular probe [^68^Ga]Ga-NOTA-COG1410 (Figure 1A) for the diagnosis of orthotopic colon cancer and the differential diagnosis of orthotopic colon cancer and colitis in mice [13]. Micro-PET/CT showed high uptake of [^68^Ga]Ga-NOTA-COG1410 in tumors but no uptake in enteritis 0.5 h after tail vein injection. However, [^18^F]FDG micro-PET/CT showed high uptake in both enteritis and tumors. [^68^Ga]Ga-NOTA-COG1410 had potential in the differentiation of colon cancer and colitis but showed high uptake in the liver, thus affecting the exploration of metastases in this organ. Shi et al. synthesized [^68^Ga]Ga-NOTA-PEG_11_-COG1410 with the aim of reducing the uptake of tracers in the liver by increasing hydrophilicity, but this could not be achieved, so they instead targeted TREM2 through radioimmunoimaging. Shi et al. prepared a TREM2-targeting clonal antibody 5-mAb by immunizing mice with TREM2 antigen and prepared 5-F(ab′)_2_ and 5-Fab antibody fragments. Together with the purchased mabs (clone 237920), six radioimmune probes were finally synthesized (Figure 1B): [^124^I]I-5-mAb, [^124^I]I-5-F(ab′)_2_, [^124^I]I-anti-TREM2-mAb, [^124^I]I-anti-TREM2-F(ab′)_2_, [^99m^Tc]Tc-MAG_3_-5-F(ab′)_2_ and [^99m^Tc]Tc-MAG_3_-5-Fab [14,15]. A comparative study revealed that [^124^I]I-5-F(ab′)_2_ had higher uptake in the tumor site but lower uptake in other tissues and organs, thus showing potential for the diagnosis and staging of tumors. For neuroimaging, Meier et al. presented a ^124^I-labeled TREM2 antibody with a linked single-chain variable fragment that binds the transferrin receptor to target TREM2 in AD mice. Using PET, the tracer was unable to image TREM2 in vivo but could visualize TREM2 ex vivo [16].

## 3. CSF1R

**Figure 2:**
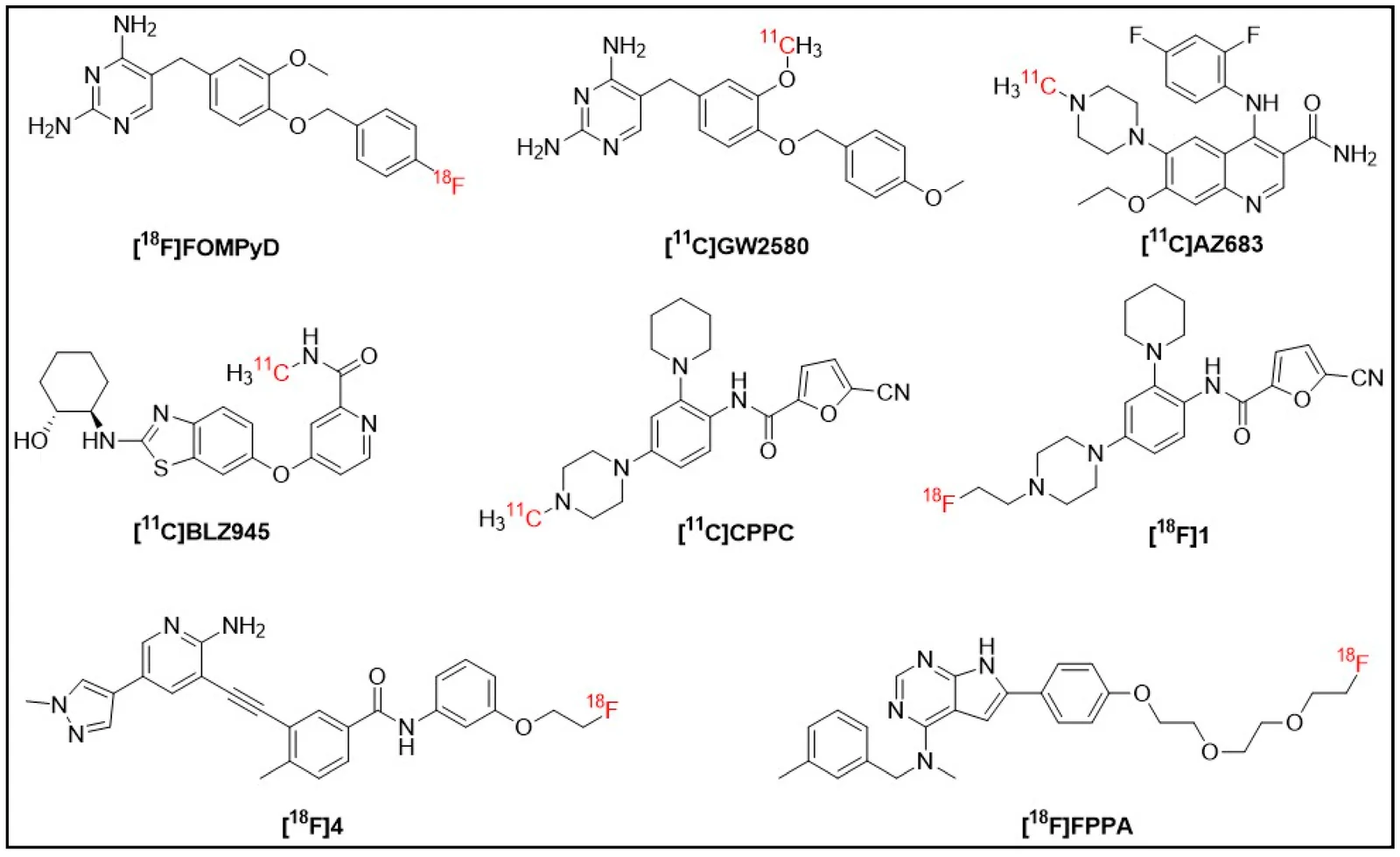
The molecular formulas of reported PET tracers targeting CSF1R.

Imaging tracers targeting colony-stimulating factor 1 receptor (CSF1R) were developed in a short period; no imaging tracer targeting TAMs has yet been reported, but it still has the potential to target TAMs [17]. CSF1R is a tyrosine kinase transmembrane receptor that belongs to the growth factor CSF-1/platelet-derived growth factor (PDGF) receptor family [17]. In the brain, CSF1R is specifically expressed in microglia. Extracranially, CSF1R is mainly expressed in monocytes/macrophages [18,19]. The ligands of CSF1R include colony-stimulating factor 1 (CSF1) and IL-34. When CSF1R binds CSF1 or IL-34, it needs to dimerize and bind ATP to activate downstream signaling pathways to regulate the polarization of microglia/macrophages, promoting their polarization toward an anti-inflammatory, pro-tumor M2-type phenotype. CSF1R inhibitors regulate the tumor microenvironment by inhibiting the downstream signaling pathway of CSF1R and promote the polarization of TAMs from tumor-promoting M2-type to anti-tumor M1-type macrophages, thus playing an anti-tumor role [20]. The molecular formulas of reported PET tracers targeting CSF1R are exhibited in Figure 2. The first reported CSF1R PET imaging tracer is [^18^F]FOMPyD, which was modified from the CSF1R inhibitor GW2580. Autoradiography results in rat and human brain tissues indicate that it has a high level of nonspecific binding [21]. [^11^C]GW2580, another imaging tracer based on GW2580, demonstrated moderate to high brain uptake in studies with rhesus monkeys [22]. However, research indicates that both FOMPyD and GW2580 are selective inhibitors of two targets, type II myosin kinase (TrK) and CSF1R, which limits their subsequent applications [21,23,24]. The third reported CSF1R PET imaging tracer is [^11^C]AZ683. However, its low penetration and high nonspecific binding in the brains of rodents and non-human primates have limited its involvement in further research [25]. Subsequently, researchers designed [^11^C]BLZ945 based on the CSF1R inhibitor BLZ945 and found that [^11^C]BLZ945 is a substrate of efflux transporters; its high nonspecific binding in the mouse brain also limits its clinical application [26]. Currently, [^11^C]CPPC, derived from the CSF1R inhibitor CPPC (IC_50_ = 0.78 nM), is the most extensively studied CSF1R PET imaging tracer and has demonstrated high specific uptake in autoradiography studies of acute and chronic inflammatory disease mouse models, non-human primates, and postmortem AD brain tissue sections [27]. Although [^11^C]CPPC targeting CSF1R PET imaging results have shown good biological properties, its clinical application is limited due to the short half-life of ^11^C (20.4 min). To achieve a longer half-life, [^18^F]1 based on modified CPPC was successfully synthesized. Although it showed good specific binding in a mouse model of lipopolysaccharide-induced acute neuroinflammation, recent studies have confirmed that CPPC interacts with multiple kinase receptors [28]. An and colleagues synthesized [^18^F]4 [29], which possesses high specificity and in vitro stability. However, its metabolites in plasma/cerebrospinal fluid remain unknown, and it has not been evaluated using PET in animal models. In 2026, Fu et al. reported a next-generation CSF1R PET probe, [^18^F]FPPA, which exhibits excellent affinity and specificity both in vitro and in vivo, enabling clear visualization of inflammatory lesions in a rat model of neuroinflammation [30].

## 4. CD206

**Figure 3:**
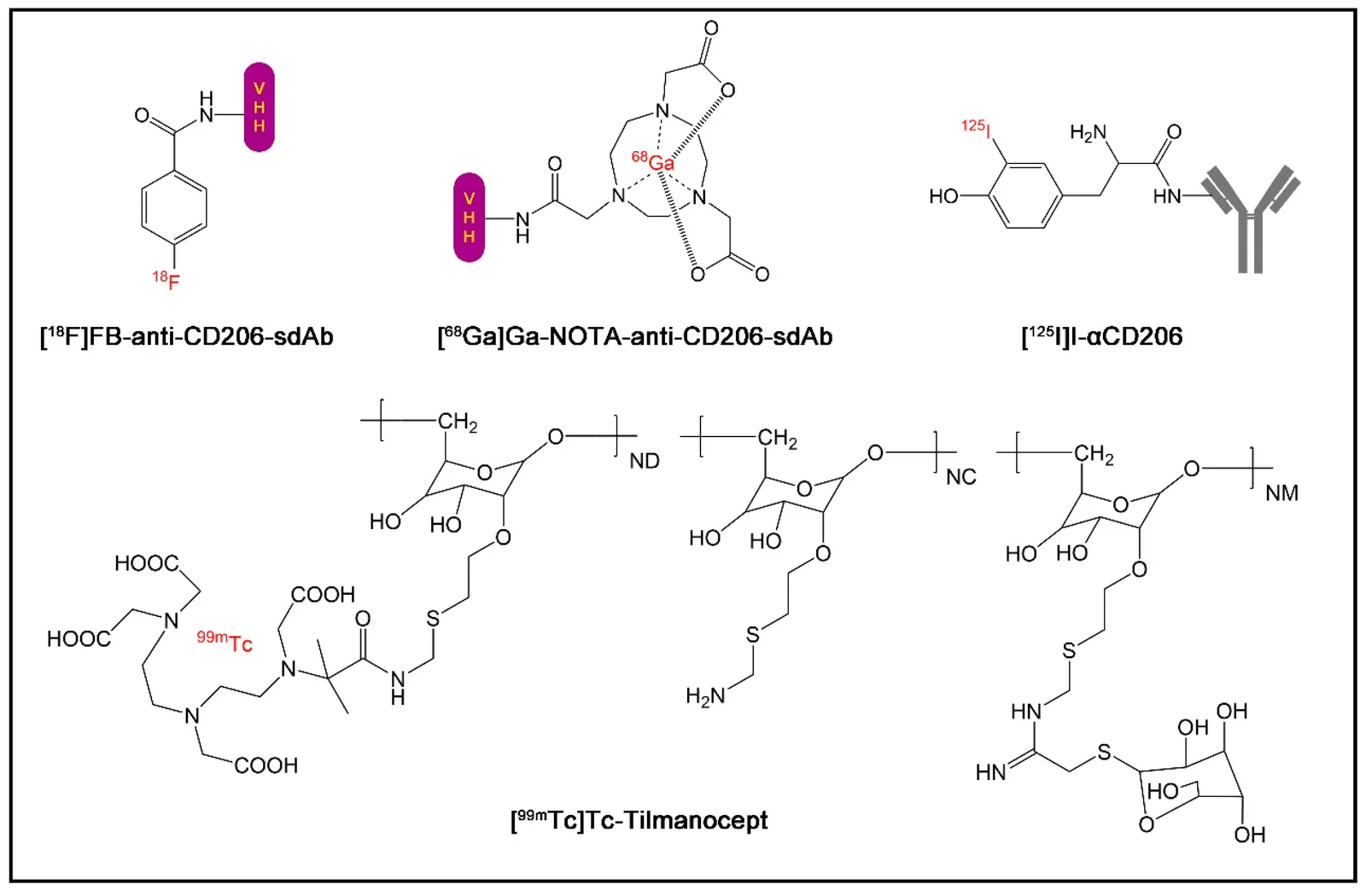
The molecular formulas of reported nuclear medicine tracers targeting CD206.

CD206, also known as the Macrophage Mannose Receptor (MMR), is a marker for TAMs [11]. MMR has multiple extracellular domains capable of recognizing various ligands, forming the basis for imaging targeted at MMR. The molecular formulas for the CD206-targeted tracers are shown in Figure 3. Regarding PET imaging tracers, Blykers et al. successfully synthesized [^18^F]FB-anti-MMR-sdAbs, guided by alpaca-derived nanobody anti-MMR sdAbs. The [^18^F]FB-anti-MMR-sdAbs exhibited high uptake in tumor regions of a mouse model of lung cancer but no uptake in an MMR-knockout mouse model [31]. Similarly, Xavier et al. synthesized [^68^Ga]Ga-NOTA-anti-MMR-sdAb, which also showed high uptake in tumor regions of a mouse model of lung cancer but no uptake in an MMR-knockout model [32]. For single-photon emission computed tomography (SPECT) imaging tracers, Zhang et al. developed a multimodal imaging tracer, [^125^I]I-αCD206, based on CD206 monoclonal antibodies combined with near-infrared fluorescence. They found that [^125^I]I-αCD206 could be used for non-invasive monitoring of tumor recurrence and lymphatic metastasis in a mouse model [33]. In clinical research by Marcinow et al., the CD206-targeted single-photon probe [^99m^Tc]Tc-Tilmanocept was shown to aid in the localization of sentinel lymph nodes in patients with oral squamous cell carcinoma [34]. Similarly, in 2025, Boughdad et al. utilized [^99m^Tc]Tc-Tilmanocept imaging to evaluate CD206 expression in lesions of melanoma patients and demonstrated through pathological staining a strong correlation between CD206 expression and radiotracer uptake in the lesions [35]. Additionally, Movahedi et al. successfully prepared [^99m^Tc]Tc-anti-MMR Nb using histidine-tagged nanobodies and ^99m^Tc(CO)_3_(H_2_O)_3_, indicating that this probe could non-invasively assess the distribution of TAMs [36].

## 5. CXCR4

**Figure 4:**
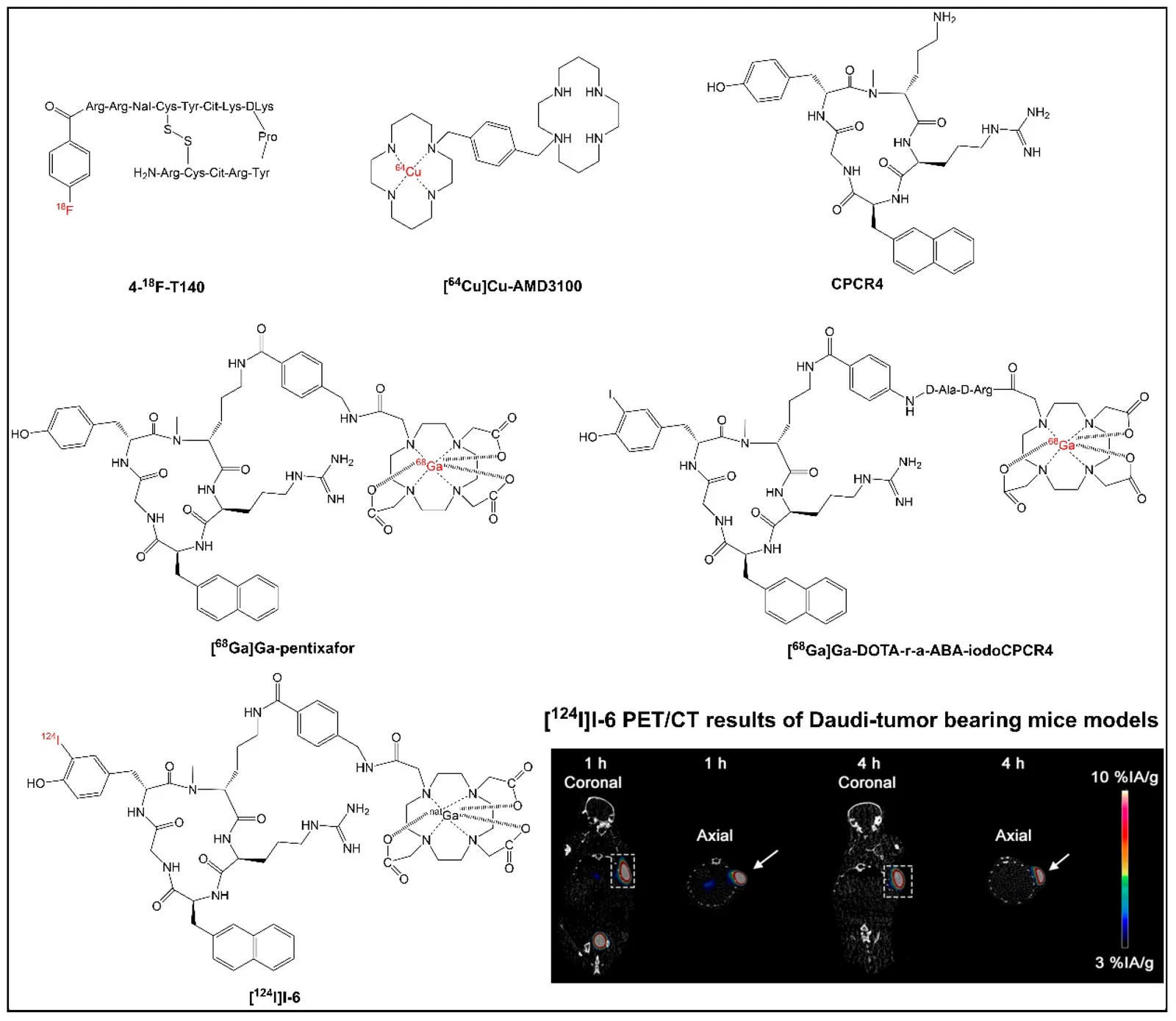
The molecular formulas of reported nuclear medicine tracers targeting CXCR4. Reproduced with permission from Dai Shi, 2024 [46].

Chemokine (C-X-C motif) receptor 4 (CXCR4) is a G-protein-coupled receptor (GPCR) with seven transmembrane structures composed of 352 amino acids involved in regulating important physiological mechanisms in the body [37,38]. Overactivation of CXCR4 has been demonstrated in TAMs, which provides a molecular and biological basis for the integrated diagnosis and treatment of tumors with high CXCR4 expression [37,38]. The molecular formulas of reported nuclear medicine tracers targeting CXCR4 are shown in Figure 4. [^64^Cu]Cu-AMD3100 and 4-^18^F-T140 were the first-generation PET imaging tracers that targeted CXCR4 [39,40] (Figure 4). The high abdominal background of 4-^18^F-T140 and [^64^Cu]Cu-AMD3100 limited their widespread application. Among the second-generation probes targeting CXCR4, pentixafor, designed with CPCR4 (cyclo (-D-Tyr-n-me-d-Orn-L-Arg-L-2-NAL-Gly-)) as the pharmacophore, undoubtedly stands out as the brightest star [41]. [^68^Ga]Ga-pentixafor not only had the potential for CXCR4 assessment in malignant tumors but also demonstrated higher diagnostic specificity and sensitivity than FDG in the diagnosis of primary aldosteronism and pheochromocytoma [42,43,44]. [^68^Ga]Ga-pentixafor is currently the most widely used PET probe targeting CXCR4. Although [^68^Ga]Ga-pentixafor exhibited excellent physicochemical properties, its affinity significantly decreased after changing the radionuclide [41]. Therefore, third-generation molecular tracers targeting CXCR4 have been developed and are increasingly being used in theranostic applications. Osl et al. synthesized [^68^Ga]Ga-DOTA-r-a-ABA-iodoCPCR4 using iodoCPCR4 as the pharmacophore, introducing several amino acid linkers between the pharmacophore and the chelator. Similar to [^68^Ga]-pentixafor, [^68^Ga]Ga-DOTA-r-a-ABA-iodoCPCR4 exhibited excellent target-to-background ratios. However, the affinity of [^68^Ga]Ga-DOTA-r-a-ABA-iodoCPCR4 remained unaffected after changing the radiolabel, indicating potential for theranostic applications [45]. Cheng et al. synthesized six PET/SPECT molecular probes targeting CXCR4, employing CPCR4 as the pharmacophore [46]. Comparative studies revealed that even subtle structural modifications to probes with the CPCR4 pharmacophore could significantly alter their affinity. Ultimately, they found [^124^I]I-6 to possess the optimal image-to-background ratio. More importantly, when replacing iodine-124 with iodine-131, it still exhibited excellent target-to-background ratios and achieved remarkable therapeutic efficacy in animal models. [^124^I]I-6 and [^131^I]I-6 are currently considered a promising theranostic probe combination. It should be noted that in the above-mentioned studies, it remains impossible to distinguish whether the high uptake of imaging tracers in tumors primarily originates from tumor cells or TAMs, as CXCR4 expression lacks the specificity exhibited by CD206 and TREM2.

## 6. Folate Receptors

**Figure 5:**
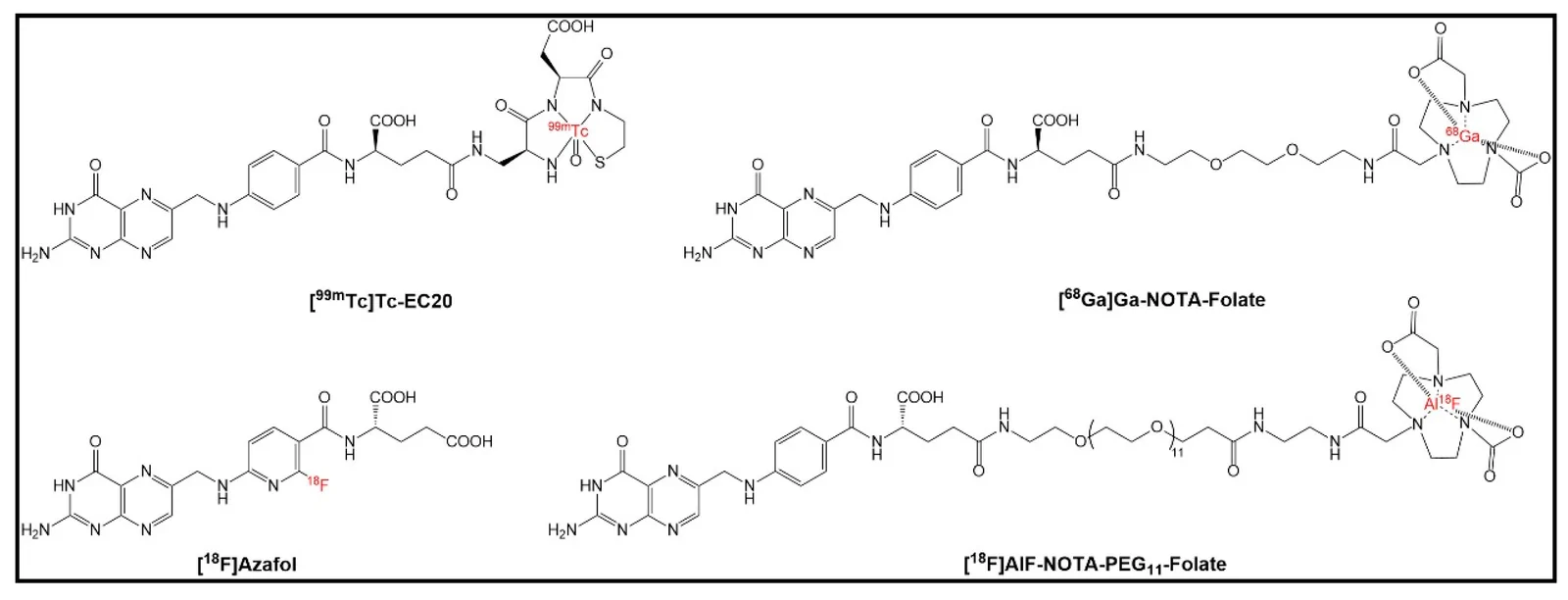
The molecular formulas of reported nuclear medicine tracers targeting folate receptors.

Folate receptors are a type of N-glycosylated protein, and they are primarily classified into four subtypes: FRα, FRβ, FRγ, and FRδ [47]. Among them, folate receptor FRβ is highly expressed in TAMs, whereas FRα is predominantly expressed on tumor cells and only minimally expressed in TAMs [48]. The folate receptor-targeted imaging tracers introduced in this section were developed based on the same pharmacophore. Constrained by the specificity of this pharmacophore, these probes bind to both FRα and FRβ. Therefore, it should be noted that current folate receptor imaging cannot precisely determine whether the tumor signal originates primarily from TAMs or from the tumor cells themselves. Brand et al. conducted a comparative study of two folate receptor-targeting imaging tracers, [^99m^Tc]Tc-EC20 and [^68^Ga]Ga-NOTA-Folate [49]. The study revealed that [^68^Ga]Ga-NOTA-Folate exhibited a longer tumor retention time compared to [^99m^Tc]Tc-EC20 in KB tumor-bearing mouse models. Gnesin et al. conducted clinical trials with [^18^F]AzaFol, demonstrating that it offers a reasonable radiation dose and provides clear visualization of tumors [50]. Additionally, [^18^F]AzaFol can cross the blood–brain barrier, suggesting its potential for imaging brain tumors or metastases. Chen et al. synthesized [^18^F]AlF-NOTA-PEG_11_-Folate. Compared to [^18^F]AlF-NOTA-Folate, [^18^F]AlF-NOTA-PEG_11_-Folate incorporates PEG_11_ units between the folate and NOTA moieties [51]. This modification resulted in reduced hepatic accumulation without compromising tumor uptake, significantly enhancing the contrast of liver metastases. The molecular structures of folate receptor-targeting imaging tracers are depicted in Figure 5.

## 7. Arginase

**Figure 6:**
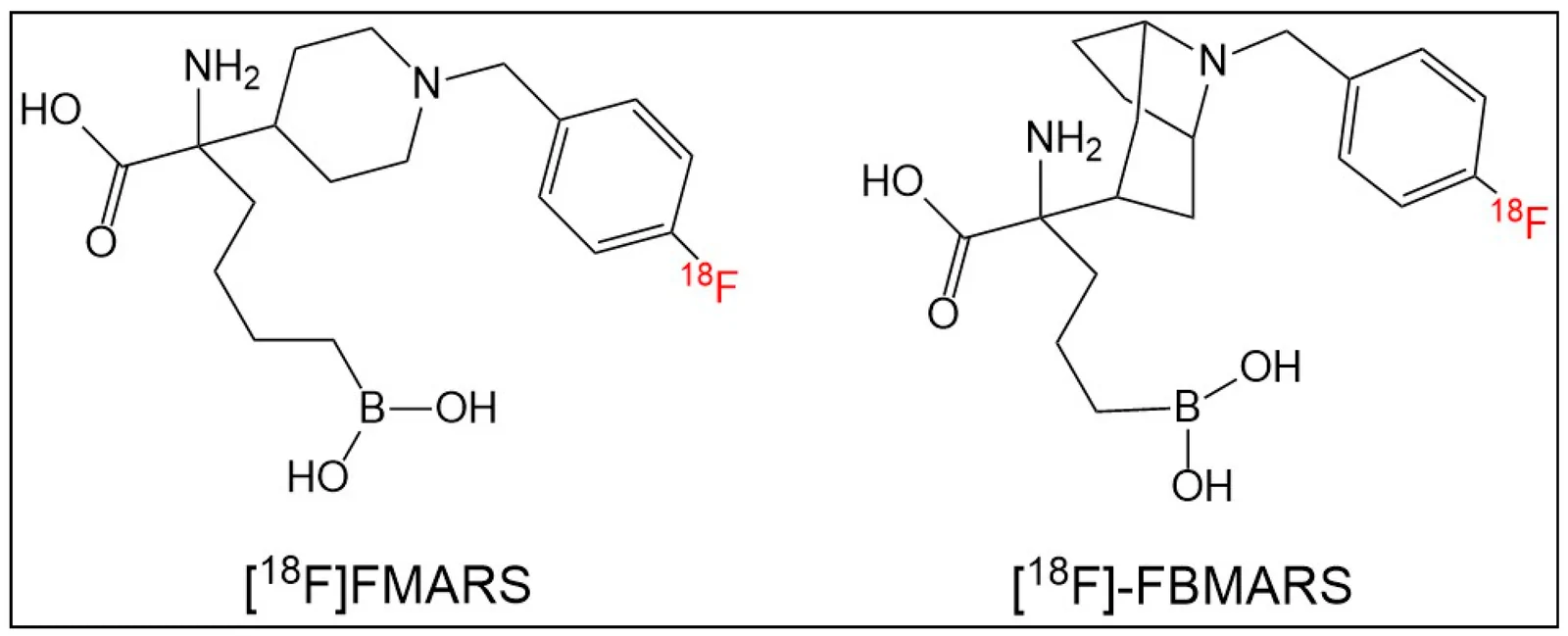
The molecular formulas of [^18^F]FMARS and [^18^F]FBMARS.

Arginase is an enzyme that catalyzes the hydrolysis of L-arginine into ornithine and urea. In the tumor microenvironment, the expression of arginase is significantly upregulated in myeloid cells, specifically TAMs [52]. This biological alteration provides a foundation for targeting TAMs for tumor imaging. Clemente et al. synthesized [^18^F]FMARS and [^18^F]FBMARS, two arginase-targeting tracers [52]. Comparative studies demonstrated that [^18^F]FBMARS exhibits greater uptake in mouse prostate cancer xenografts and higher affinity than [^18^F]FMARS. The molecular structures of [^18^F]FMARS and [^18^F]FBMARS are depicted in Figure 6. It is important to emphasize that certain tumors also exhibit high expression of arginase, and it remains challenging to accurately distinguish whether the PET signal originates from tumor cells or TAMs.

## 8. M2 Macrophage-Binding Peptide (Ligand)

**Figure 7:**
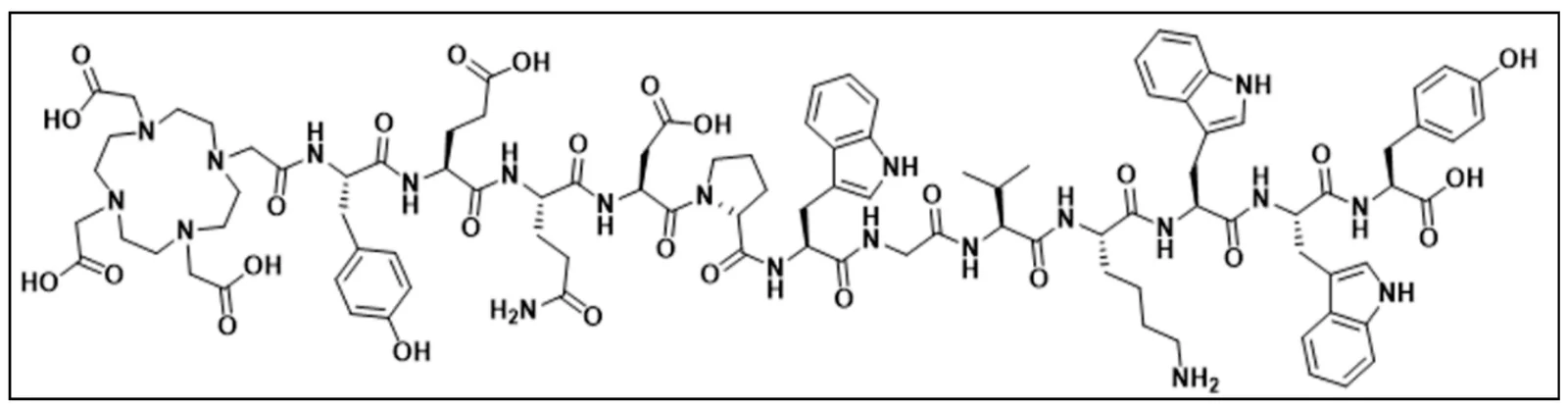
The molecular formula of DOTA-M2pep.

M2-type macrophage-binding peptide (M2pep) exhibits high selectivity and affinity for targeting M2-type macrophages. Studies have shown that M2pep can be used in drug delivery systems targeting tumor-associated macrophages, resulting in promising therapeutic outcomes [53]. Huang et al. reported a ^68^Ga-labeled peptide molecular probe, ^68^Ga-DOTA-M2pep [54], designed based on M2pep. This probe demonstrated excellent specificity both in vitro and in vivo. Huang performed a correlation analysis of results from small-animal PET and slice immunofluorescence, revealing that ^68^Ga-DOTA-M2pep can dynamically reflect the distribution of tumor-associated macrophages in mouse tumor models. The molecular structure of DOTA-M2pep is shown in Figure 7.

## 9. rHDL (Ligand)

TAMs in the tumor microenvironment typically exhibit a “lipid-rich” or “foam cell-like” metabolic state, characterized by high expression of scavenger receptors (Scavenger Receptor Class B Type 1). rHDL closely mimics native human high-density lipoprotein particles in both structure and composition. Upon entering the body, rHDL is recognized by these receptors on the surface of TAMs and is specifically taken up and retained intracellularly via receptor-mediated endocytosis. Research has confirmed that rHDL is specifically taken up by macrophages [55]. Pérez-Medina and his colleagues developed two positron-emitting molecular probes based on rHDL: [^89^Zr]Zr-AI-HDL and [^89^Zr]Zr-PL-HDL [56]. Through in vivo and in vitro assessments, they demonstrated that these probes can be used for non-invasive monitoring of TAM distribution in mouse models of breast cancer. However, due to the large molecular weight of the probes themselves, it exhibits extremely high accumulation in the liver and intestines, which limits their further clinical application.

## 10. B7–H4

B7–H4, a type I transmembrane glycoprotein, is a member of the B7/CD28 family. It is highly expressed in TAMs, and its overexpression is particularly relevant in prostate cancer, where it correlates with an aggressive tumor phenotype, higher Gleason scores, and advanced disease stages [57]. Kumar et al. employed a monoclonal antibody as the targeting moiety to develop [^89^Zr]Zr-DFO-2H9, a B7-H4-targeted immune-PET probe. Exhibiting high specificity and affinity, this probe enables the in vivo monitoring of the dynamic distribution of TAMs in prostate cancer models [58]. Similarly, the further clinical translation of this [^89^Zr]Zr-DFO-2H9 is hindered by its large molecular weight, which results in slow clearance and high hepatic background.

## 11. Discussion

Imaging TAMs holds promise in differentiating between tumors and inflammation, as well as in tumor monitoring. Firstly, macrophages infiltrating the tumor immune microenvironment are known as TAMs, which predominantly exhibit an M2-type macrophage phenotype. M2-type macrophages are anti-inflammatory and pro-tumorigenic, while M1-type macrophages primarily reside in inflammatory lesions, thus promoting inflammation and inhibiting tumor growth. Due to the distinct expression patterns of surface receptors on different macrophage subtypes, PET imaging tracers targeting TAMs or M1 macrophages can be utilized for differential diagnosis between tumors and inflammatory lesions. For instance, ^68^Ga-NOTA-COG1410 targeting the TREM2 receptor (biomarker of TAMs) exhibited high uptake in colon cancer lesions but low uptake in colitis [13]. Secondly, owing to the significant proportion of TAMs within tumor tissues, TAM-targeted imaging tracers also possess the potential for broad-spectrum tumor diagnosis. Achieving broad-spectrum tumor diagnosis requires identifying targets that exhibit substantial expression differences between tumor tissues and normal tissues. TAMs originate from monocytes, while “cancer” originates from epithelial tissues. The expression levels of receptors on cells/tissues with different origins tend to differ significantly, such as TREM2 and CD206. Therefore, TAM-targeted imaging holds clinical potential for tumor diagnosis, monitoring, staging, and differential diagnosis. For example, [^68^Ga]Ga-Anti-CD206-sdAb is currently undergoing clinical trials in solid tumors and has shown promising potential for application [59].

Beyond its potential in imaging, targeting TAMs for immunotherapy also shows promise. In recent years, immunotherapy has benefited many cancer patients. The current focus in clinical cancer immunotherapy is PD-1/PD-L1 therapy targeting T cells. Numerous studies have demonstrated that TAMs contribute to tumor cell immune evasion [60,61]. Therefore, immunotherapy targeting TAMs has emerged as a major area of research. Kuang et al. discovered that the high expression of PD-L1 on TAMs could be the “culprit” responsible for immune escape [62]. Gordon et al. found that anti-PD1 treatment enhanced the phagocytic ability of TAMs, significantly extending the survival time of colon cancer models [60]. Binnewies et al. discovered that in mouse syngeneic tumor models and human solid tumors of various histological types, TREM2^+^ TAMs are associated with CD8^+^ tumor-infiltrating lymphocyte (TIL) depletion, and anti-TREM2-mAb monoclonal antibody treatment significantly enhances the efficacy of tumor immunotherapy [63]. Several drugs targeting TAMs are currently entering clinical trials, such as inhibitors targeting CXCR4 and CSF1R [64]. Taking CSF1R as an example, besides the clinically approved drugs pexidartinib and surufatinib, there are numerous CSF1R inhibitors currently in clinical trials. Inhibitors like emactuzumab, pimicotinib, and vimseltinib are in Phase III clinical trials, while cabiralizumab and axatilimab are in Phase II clinical trials. These trials involve various cancer types, including lung cancer, breast cancer, pancreatic cancer, prostate cancer, ovarian cancer, renal cancer, and giant cell tumor of the tendon sheath, and have shown promising therapeutic effects. Therefore, assessing the expression level of a specific target on TAMs may provide guidance for the use of TAM-targeted immunotherapy in the future. Additionally, target evaluation through nuclear medicine PET/SPECT imaging can help mitigate the spatial and temporal heterogeneity of target expression to some extent.

This review summarized the imaging targets of TAMs and their corresponding nuclear medicine tracers (Table 1 and Table 2). In Table 1, we summarize all the tracers reviewed in this manuscript, categorized by their respective targets. For each entry, the target, radiotracer, species, disease (model), strengths, limitations, and corresponding references are provided. In Table 2, we provide a cross-comparison of the general characteristics, advantages, and disadvantages of each target. Notably, although this review includes at least eight targets highly expressed in TAMs, only a few exhibit high expression specificity: TREM2, CD206, and CSF1R. Therefore, more highly specific targets need to be identified to further expand the repertoire of TAM-targeted imaging.

Despite the promising preclinical and early clinical data, several translational hurdles must be addressed before TAM-targeted tracers can be routinely implemented in clinical practice. First, scalable and reproducible tracer production under current Good Manufacturing Practice (cGMP) conditions remains a bottleneck, particularly for antibody- or nanobody-based tracers that require complex bioconjugation and radiolabeling procedures. Second, comprehensive pharmacokinetic profiling and radiation dosimetry studies in humans are still lacking for most candidates; without these data, regulatory agencies cannot adequately assess safety or establish optimal imaging windows. Third, even when tumor uptake is favorable, high background signal in non-target organs (e.g., liver and spleen) may compromise diagnostic accuracy and limit the detection of metastatic lesions. Furthermore, regulatory approval pathways for novel TAM-targeted imaging tracers are not yet well defined, as companion diagnostic criteria and standardized image interpretation protocols have not been established. Finally, integration into routine clinical workflows will require validation through multicenter trials demonstrating added value over existing standards such as [^18^F]FDG PET/CT, as well as cost-effectiveness analyses to justify reimbursement. Addressing these challenges through coordinated efforts among academia, industry, and regulators will be essential to realize the full clinical potential of TAM-targeted nuclear medicine imaging.

In addition to the challenges of clinical translation, TAM-targeted nuclear medicine imaging also faces biological “challenges”. Emerging single-cell RNA sequencing and spatial transcriptomics studies are reshaping our understanding of TAMs’ heterogeneity, revealing a multidimensional activation continuum that extends far beyond the classical M1/M2 binary framework. An authoritative study has demonstrated that TAMs can undergo dynamic reprogramming in response to therapeutic interventions [65]. For instance, certain treatments may induce a shift from an immunosuppressive transcriptional program toward an immunostimulatory one without completely abolishing the expression of canonical M2 markers, potentially leading to misinterpretation of imaging signals. Therefore, future probe development may need to focus not only on the expression levels of surface antigens on TAMs but also on the dynamic evolution of TAMs within the lesion.

## 12. Future Outlook

Currently, the most promising research direction in nuclear medicine is precision theranostics. The first generation of nuclear medicine theranostics is exemplified by iodine-131 (^131^I) therapy for hyperthyroidism. The “diagnostic” component involves measuring radioiodine uptake to aid in diagnosis and calculate the administered activity, while the “therapeutic” component entails oral administration of ^131^I to ablate hyperfunctioning thyroid tissue. The second generation is represented by radioimmunoimaging and radioimmunotherapy, which utilize monoclonal antibodies or antibody fragments as targeting moieties. These molecules offer high specificity and affinity; however, their relatively large molecular weights (e.g., ~150 kDa for IgG) result in slow blood clearance and high background uptake in non-target tissues [66], severely limiting clinical application. In recent years, nanobodies (~15 kDa) have demonstrated promising theranostic potential, exhibiting excellent specificity and affinity with significantly faster blood clearance than conventional IgG antibodies [67]. Nevertheless, the prolonged renal retention of nanobodies poses challenges to kidney safety. The third generation is characterized by peptide receptor-mediated radionuclide therapy, with [^177^Lu]Lu-PSMA-617 as the most representative example. Peptide-based molecular probes offer multiple advantages, including ease of synthesis, good batch-to-batch consistency, low immunogenicity, high specificity, and rapid clearance.

Traditional radiopharmaceutical development relies heavily on high-throughput screening and extensive trial-and-error experimentation to identify candidates with favorable physicochemical and biological properties. This approach is characterized by prolonged development cycles, high costs, and low success rates, struggling to precisely meet the unique requirements of radiopharmaceuticals such as radionuclide labeling compatibility and targeting specificity. These limitations have become a critical bottleneck constraining field advancement. Artificial intelligence-assisted drug design is now driving a paradigm shift in radiopharmaceutical R&D, moving from a trial-and-error-driven model toward an intelligent design approach.

First, deep learning-based molecular generative models (e.g., variational autoencoders, generative adversarial networks, and diffusion models) can rapidly explore vast chemical spaces to propose novel small-molecule or peptide scaffolds with optimized binding affinity for specific TAM-associated receptors, dramatically accelerating hit identification [68,69]. Second, structure-based virtual screening augmented by graph neural networks and transformer-based protein–ligand interaction predictors enables accurate docking and scoring against targets such as CSF1R and TREM2, even in the absence of high-resolution crystal structures, through the integration of AlphaFold2/3-predicted receptor conformations [70]. Third, machine learning models trained on large pharmacokinetic datasets can predict and optimize critical radiopharmaceutical parameters—including lipophilicity (logP), plasma protein binding, renal versus hepatobiliary clearance, and metabolic stability—thereby improving tumor-to-background ratios and reducing off-target radiation dose to normal organs [71]. This is particularly relevant given that several TAM-associated receptors (e.g., CD206 in the liver and spleen and CXCR4 in bone marrow and kidneys) are also expressed in healthy tissues, necessitating exquisite selectivity engineering.

Looking ahead, AI-driven multi-objective optimization frameworks can simultaneously balance radionuclide labeling compatibility (e.g., coordination geometry for ^68^Ga, ^177^Lu, or ^225^Ac chelation), receptor-binding affinity, internalization kinetics, and in vivo stability—parameters that are notoriously difficult to co-optimize through empirical methods.

## Figures and Tables

**Table 1:** Summary of potential PET/SPECT tracers for TAMs, evaluated in preclinical or clinical studies.

Targets	Radiotracer	Species	Disease (Model)	Strengths	Limitations	References
TREM2	[^68^Ga]Ga-NOTA-COG1410	mouse	colon cancer in situ	rapid blood clearance; differential diagnosis of colon cancer and colitis	high non-specific hepatic uptake	[13]
	[^124^I]I-anti-TREM2-mAb	human	gastric cancer	high specificity	slow blood clearance rate	[14]
	[^124^I]I-anti-TREM2-F(ab′)_2_	human	gastric cancer	high specificity	moderate blood clearance rate	[14]
	[^124^I]I-5-mAb	human	gastric cancer	high specificity; high affinity	slow blood clearance rate	[14]
	[^124^I]I-5-F(ab′)_2_	human	gastric cancer	high specificity; high affinity	moderate blood clearance rate	[14]
	[^99m^Tc]Tc-MAG_3_-5-F(ab′)_2_	human	lung cancer	high specificity; high affinity	moderate blood clearance rate	[15]
	[^99m^Tc]Tc-MAG_3_-5-Fab	human	lung cancer	high specificity; rapid blood clearance	enterohepatic circulation	[15]
CSF1R	[^18^F]FOMPyD	rat/human	wild-type rats/human brain tissues	rapid blood clearance	low specificity	[21]
	[^11^C]GW2580	mouse/rhesus monkey	mouse models of acute and chronic neuroinflammation/healthy rhesus monkey	rapid blood clearance; moderate to high brain uptake	excessively short physical half-life	[22]
	[^11^C]AZ683	rat/rhesus monkey	wild-type rats/healthy rhesus monkey	rapid blood clearance	excessively short physical half-life; low penetration	[25]
	[^11^C]BLZ945	mouse	wild-type mouse	rapid blood clearance	excessively short physical half-life; substrate of efflux transporters	[26]
	[^11^C]CPPC	mouse	AD	rapid blood clearance	excessively short physical half-life	[27]
	[^18^F]1	mouse	neuroinflammation	rapid blood clearance	low specificity	[28]
	[^18^F]4	/	/	/	/	[29]
	[^18^F]FPPA	rat	neuroinflammation	rapid blood clearance	low specificity	[30]
CD206	[^18^F]FB-anti-MMR-sdAbs	mouse	lung cancer	high specificity	prolonged renal retention	[31]
	[^68^Ga]Ga-NOTA-anti-MMR-sdAb	mouse	lung cancer	high specificity	prolonged renal retention	[32]
	[^125^I]I-αCD206	mouse	breast cancer	high specificity	slow blood clearance rate	[33]
	[^99m^Tc]Tc-Tilmanocept	human	patients with oral squamous cell carcinoma	High negative predictive value; low false-negative rate	study of lymph node metastases only	[34,35]
	[^99m^Tc]Tc-Nanobody	mouse	lung cancer	high specificity	prolonged renal retention	[36]
CXCR4	[^64^Cu]Cu-AMD3100	mouse	wild-type mouse	rapid blood clearance	high radioactive distribution in the liver and intestines; low specificity	[39]
	4-^18^F-T140	human	ovarian cancer	rapid blood clearance	high radioactive distribution in the liver and intestines; low specificity	[40]
	[^68^Ga]Ga-pentixafor	human	lung cancer	high affinity; rapid blood clearance	fixed compound structure	[41]
	[^68^Ga]Ga-DOTA-r-a-ABA-iodoCPCR4	human	lymphoma	high affinity; rapid blood clearance	complex synthetic route	[45]
	[^124^I]I-6	human	lymphoma	high affinity; rapid blood clearance; used for theranostics	Iodine-124 is difficult to obtain	[46]
Folate receptors	[^99m^Tc]Tc-EC20	human	oral squamous cell carcinoma	rapid blood clearance	short tumor retention time	[49]
	[^68^Ga]Ga-NOTA-Folate	human	oral squamous cell carcinoma	rapid blood clearance; long tumor retention time	/	[49]
	[^18^F]AzaFol	human	patients with lung cancer	rapid blood clearance; clinical studies; cross the blood–brain barrier	high non-specific hepatic uptake	[50]
	[^18^F]AlF-NOTA-PEG_11_-Folate	human	oral squamous cell carcinoma/lung cancer	rapid blood clearance; low non-specific hepatic uptake	low specificity	[51]
Arginase	[^18^F]FMARS	human	prostate cancer	rapid blood clearance	lower affinity; high non-specific hepatic uptake	[52]
	[^18^F]FBMARS	human	prostate cancer	higher affinity	high non-specific hepatic uptake	[52]
M2 macrophage-binding peptide	[^68^Ga]Ga-DOTA-M2pep	mouse	melanoma	rapid blood clearance	unclear binding target; low specificity	[54]
rHDL	[^89^Zr]Zr-AI-HDL	mouse	breast cancer	High tumor uptake	slow blood clearance rate; poor stability	[56]
	[^89^Zr]Zr-PL-HDL	mouse	breast cancer	High tumor uptake	slow blood clearance rate	[56]
B7–H4	[^89^Zr]Zr-DFO-2H9	human	prostate cancer	High tumor uptake	slow blood clearance rate; high non-specific hepatic uptake	[58]

**Table 2:** Comparative analysis of key TAM-associated targets for nuclear medicine imaging.

Targets	Biological Specificity	Relative Advantages	Limitations/Challenges	Suitable Cancer Types
TREM2	High specificity for TAMs; minimal expression on steady-state tissue-resident macrophages	Excellent marker for TAMs; correlates with T-cell exclusion and poor prognosis	Complex biology not fully understood	Significantly upregulated in nearly all solid malignancies
CD206	M2-like phenotype marker; also expressed on liver sinusoidal endothelial cells & dendritic cells	Excellent marker for TAMs; abundant expression facilitates high signal-to-noise ratio	Limited specificity (liver/spleen background)	Angiogenesis-driven tumors; Most solid malignant tumors
CSF1R	Expressed on monocytes, macrophages, and dendritic cells	Broad TAMs coverage	Lower specificity than TREM2 and CD206 due to expression on circulating monocytes	Current research focused on central nervous system disorders, with no studies evaluating its application in oncology to date
CXCR4	Broad chemokine receptor; expressed on TAMs, T cells, and tumor cells	Established clinical tracers available; high expression level in tumors	Low specificity for TAMs alone	Significantly overexpressed in more than 20 types of malignant tumors
Folate Receptor β	Restricted to activated (M2-like) macrophages; absent on resting macrophages	Minimal background in normal tissues	Currently available tracers lack sufficient specificity	Oral squamous cell carcinoma and NSCLC with high FRβ+ TAM infiltration
Arginase-1	Intracellular enzyme; M2 polarization marker	Functional readout of immunosuppressive activity	Requires intracellular penetration	Prostate cancer
rHDL	Nanoparticle platform mimicking native HDL; targets Scavenger Receptor Class B Type 1 of TAMs	Versatile payload delivery (radionuclides, drugs); exploits natural HDL-TAM interaction	Non-specific uptake in liver and steroidogenic tissues; indirect TAM targeting	Solid tumors with dense TAM infiltration (e.g., breast)
B7–H4	Immune checkpoint molecule; highly restricted to TAMs and certain tumor cells; negligible expression on normal peripheral tissues	Dual target for both TAM imaging and immunotherapy; stable membrane expression	Heterogeneous expression across tumor types; limited validated radiotracers in clinical trials	Prostate cancer with high B7-H4^+^ TAM density

## Data Availability

No new data were created or analyzed in this study. Data sharing is not applicable to this article.

## References

[1] Mankoff D.A., Katz S.I. (2018). PET imaging for assessing tumor response to therapy. J. Surg. Oncol..

[2] Gray B.R., Agarwal A., Tann M., Koontz N.A. (2020). PET and SPECT Imaging of Brain Neoplasia Mimics. Semin. Ultrasound CT MRI.

[3] van der Geest K.S.M., Treglia G., Glaudemans A.W.J.M., Brouwer E., Sandovici M., Jamar F., Gheysens O., Slart R.H.J.A. (2021). Diagnostic value of [^18^F]FDG-PET/CT for treatment monitoring in large vessel vasculitis: A systematic review and meta-analysis. Eur. J. Nucl. Med. Mol. Imaging.

[4] Paez D., Giammarile F., Brink A., García-Pérez O., Estrada-Lobato E. (2024). The Role of 18-Fluorodeoxyglucose Positron Emission Tomography/Computed Tomography (^18^F-FDG PET/CT) in the Diagnosis and Evaluation of Spondylodiscitis. Semin. Nucl. Med..

[5] Thorsson V., Gibbs D.L., Brown S.D., Wolf D., Bortone D.S., Ou Yang T.H., Porta-Pardo E., Gao G.F., Plaisier C.L., Eddy J.A. (2018). The Immune Landscape of Cancer. Immunity.

[6] Yunna C., Mengru H., Lei W., Weidong C. (2020). Macrophage M1/M2 polarization. Eur. J. Pharmacol..

[7] Wu K., Yuan Y., Yu H., Dai X., Wang S., Sun Z., Wang F., Fei H., Lin Q., Jiang H. (2020). The gut microbial metabolite trimethylamine N-oxide aggravates GVHD by inducing M1 macrophage polarization in mice. Blood.

[8] Gunassekaran G.R., Vadevoo S.M.P., Baek M.-C., Lee B. (2021). M1 macrophage exosomes engineered to foster M1 polarization and target the IL-4 receptor inhibit tumor growth by reprogramming tumor-associated macrophages into M1-like macrophages. Biomaterials.

[9] Mantovani A., Sozzani S., Locati M., Allavena P., Sica A. (2002). Macrophage polarization: Tumor-associated macrophages as a paradigm for polarized M2 mononuclear phagocytes. Trends Immunol..

[10] Sullivan K.M., Li H., Yang A., Zhang Z., Munoz R.R., Mahuron K.M., Yuan Y.-C., Paz I.B., Von Hoff D., Han H. (2024). Tumor and Peritoneum-Associated Macrophage Gene Signature as a Novel Molecular Biomarker in Gastric Cancer. Int. J. Mol. Sci..

[11] Mantovani A., Allavena P., Marchesi F., Garlanda C. (2022). Macrophages as tools and targets in cancer therapy. Nat. Rev. Drug Discov..

[12] Blum L., Geisslinger G., Parnham M.J., Grünweller A., Schiffmann S. (2020). Natural antiviral compound silvestrol modulates human monocyte-derived macrophages and dendritic cells. J. Cell. Mol. Med..

[13] Shi D., Si Z., Xu Z., Cheng Y., Lin Q., Fu Z., Fu W., Yang T., Shi H., Cheng D. (2022). Synthesis and Evaluation of ^68^Ga-NOTA-COG1410 Targeting to TREM2 of TAMs as a Specific PET Probe for Digestive Tumor Diagnosis. Anal. Chem..

[14] Shi D., Xu Z., Cheng Y., Lin Q., Si Z., Fu W., Yang T., Shi H., Cheng D. (2023). ^124^I-Labeled Immuno-PET Targeting hTREM2 for the Diagnosis of Gastric Carcinoma. Mol. Pharm..

[15] Shi D., Fu W., Tan H., Lin Q., Shi H., Cheng D. (2024). Preclinical Evaluation of ^99m^Tc-MAG_3_-5-Fab Targeting TREM2 in Lung Cancer Mouse Models: A Comparison with ^99m^Tc-MAG_3_-5-F(ab′)2. Mol. Pharm..

[16] Meier S.R., Sehlin D., Hultqvist G., Syvänen S. (2021). Pinpointing Brain TREM2 Levels in Two Mouse Models of Alzheimer’s Disease. Mol. Imaging Biol..

[17] Ries C.H., Cannarile M.A., Hoves S., Benz J., Wartha K., Runza V., Rey-Giraud F., Pradel L.P., Feuerhake F., Klaman I. (2014). Targeting Tumor-Associated Macrophages with Anti-CSF-1R Antibody Reveals a Strategy for Cancer Therapy. Cancer Cell.

[18] Adams R.C., Carter-Cusack D., Llanes G.T., Hunter C.R., Vinnakota J.M., Ruitenberg M.J., Vukovic J., Bertolino P., Chand K.K., Wixey J.A. (2024). CSF1R inhibition promotes neuroinflammation and behavioral deficits during graft-versus-host disease in mice. Blood.

[19] Altomonte S., Pike V.W. (2023). Candidate Tracers for Imaging Colony-Stimulating Factor 1 Receptor in Neuroinflammation with Positron Emission Tomography: Issues and Progress. ACS Pharmacol. Transl. Sci..

[20] Liang X., Wang C., Wang B., Liu J., Qi S., Wang A., Liu Q., Deng M., Wang L., Liu J. (2022). Discovery of Pyrrolo[2,3-d]pyrimidine derivatives as potent and selective colony stimulating factor 1 receptor kinase inhibitors. Eur. J. Med. Chem..

[21] Singleton T.A., Bdair H., Bailey J.J., Choi S., Aliaga A., Rosa-Neto P., Schirrmacher R., Bernard-Gauthier V., Kostikov A. (2020). Efficient radiosynthesis and preclinical evaluation of [^18^F]FOMPyD as a positron emission tomography tracer candidate for TrkB/C receptor imaging. J. Label. Compd. Radiopharm..

[22] Zhou X., Ji B., Seki C., Nagai Y., Minamimoto T., Fujinaga M., Zhang M.-R., Saito T., Saido T.C., Suhara T. (2021). PET imaging of colony-stimulating factor 1 receptor: A head-to-head comparison of a novel radioligand, ^11^C-GW2580, and ^11^C-CPPC, in mouse models of acute and chronic neuroinflammation and a rhesus monkey. J. Cereb. Blood Flow Metab..

[23] Bernard-Gauthier V., Schirrmacher R. (2014). 5-(4-((4-[^18^F]fluorobenzyl)oxy)-3-methoxybenzyl)pyrimidine-2,4-diamine: A selective dual inhibitor for potential PET imaging of Trk/CSF-1R. Bioorganic Med. Chem. Lett..

[24] Lei P., Li L. (2025). Neurotrophic factors as double-edged swords in osteosarcoma: Drivers of tumour growth and immune remodelling. Front. Immunol..

[25] Tanzey S.S., Shao X., Stauff J., Arteaga J., Sherman P., Scott P.J.H., Mossine A.V. (2018). Synthesis and Initial In Vivo Evaluation of [^11^C]AZ683—A Novel PET Radiotracer for Colony Stimulating Factor 1 Receptor (CSF1R). Pharmaceuticals.

[26] van der Wildt B., Miao Z., Reyes S.T., Park J.H., Klockow J.L., Zhao N., Romero A., Guo S.G., Shen B., Windhorst A.D. (2021). BLZ945 derivatives for PET imaging of colony stimulating factor-1 receptors in the brain. Nucl. Med. Biol..

[27] Horti A.G., Naik R., Foss C.A., Minn I., Misheneva V., Du Y., Wang Y., Mathews W.B., Wu Y., Hall A. (2019). PET imaging of microglia by targeting macrophage colony-stimulating factor 1 receptor (CSF1R). Proc. Natl. Acad. Sci. USA.

[28] Lee H., Park J.-H., Kim H., Woo S.-K., Choi J.Y., Lee K.-H., Choe Y.S. (2022). Synthesis and Evaluation of a ^18^F-Labeled Ligand for PET Imaging of Colony-Stimulating Factor 1 Receptor. Pharmaceuticals.

[29] An X., Wang J., Tong L., Zhang X., Fu H., Zhang J., Xie H., Huang Y., Jia H. (2023). ^18^F-Labeled o-aminopyridyl alkynyl radioligands targeting colony-stimulating factor 1 receptor for neuroinflammation imaging. Bioorganic Med. Chem..

[30] Fu Z., Lin Q., Wang Z., Su H., Hu Z., Zhang H., Sheng R., Wang C., Cheng D. (2026). Evaluation of ^18^F-FPPA as a Positron Emission Tomography Radioligand for Imaging CSF1R in LPS-Induced Rat Model of Neuroinflammation. Mol. Pharm..

[31] Blykers A., Schoonooghe S., Xavier C., D’hoe K., Laoui D., D’huyvetter M., Vaneycken I., Cleeren F., Bormans G., Heemskerk J. (2015). PET Imaging of Macrophage Mannose Receptor–Expressing Macrophages in Tumor Stroma Using ^18^F-Radiolabeled Camelid Single-Domain Antibody Fragments. J. Nucl. Med..

[32] Xavier C., Blykers A., Laoui D., Bolli E., Vaneyken I., Bridoux J., Baudhuin H., Raes G., Everaert H., Movahedi K. (2019). Clinical Translation of [^68^Ga]Ga-NOTA-anti-MMR-sdAb for PET/CT Imaging of Protumorigenic Macrophages. Mol. Imaging Biol..

[33] Zhang C., Yu X., Gao L., Zhao Y., Lai J., Lu D., Bao R., Jia B., Zhong L., Wang F. (2017). Noninvasive Imaging of CD206-Positive M2 Macrophages as an Early Biomarker for Post-Chemotherapy Tumor Relapse and Lymph Node Metastasis. Theranostics.

[34] Marcinow A.M., Hall N., Byrum E., Teknos T.N., Old M.O., Agrawal A. (2013). Use of a novel receptor-targeted (CD206) radiotracer, 99mTc-tilmanocept, and SPECT/CT for sentinel lymph node detection in oral cavity squamous cell carcinoma: Initial institutional report in an ongoing phase 3 study. JAMA Otolaryngol. Head Neck Surg..

[35] Boughdad S., Latifyan S., Schottelius M., Blue M., Tissot S., Geldhof C., Costes J., Prior J.O., Schaefer N. (2025). Utility of [99mTc]Tc-tilmanocept, an immunosuppressive macrophage functional imaging agent in melanoma patients receiving checkpoint inhibitor treatment: A feasibility study. Cancer Immunol. Immunother..

[36] Movahedi K., Schoonooghe S., Laoui D., Houbracken I., Waelput W., Breckpot K., Bouwens L., Lahoutte T., De Baetselier P., Raes G. (2012). Nanobody-based targeting of the macrophage mannose receptor for effective in vivo imaging of tumor-associated macrophages. Cancer Res..

[37] Buck A.K., Serfling S.E., Lindner T., Hänscheid H., Schirbel A., Hahner S., Fassnacht M., Einsele H., Werner R.A. (2022). CXCR4-targeted theranostics in oncology. Eur. J. Nucl. Med. Mol. Imaging.

[38] Zhou W., Guo S., Liu M., Burow M.E., Wang G. (2019). Targeting CXCL12/CXCR4 Axis in Tumor Immunotherapy. Curr. Med. Chem..

[39] Jacobson O., Weiss I.D., Szajek L., Farber J.M., Kiesewetter D.O. (2009). 64Cu-AMD3100—A novel imaging agent for targeting chemokine receptor CXCR4. Bioorganic Med. Chem..

[40] Jacobson O., Weiss I.D., Kiesewetter D.O., Farber J.M., Chen X. (2010). PET of Tumor CXCR4 Expression with 4-^18^F-T140. J. Nucl. Med..

[41] Demmer O., Gourni E., Schumacher U., Kessler H., Wester H.J. (2011). PET imaging of CXCR4 receptors in cancer by a new optimized ligand. ChemMedChem.

[42] Yin X., Ai K., Luo J., Liu W., Ma X., Zhou L., Xiang X., Su X., Wang Y., Li Y. (2024). A comparison of the performance of 68Ga-Pentixafor PET/CT versus adrenal vein sampling for subtype diagnosis in primary aldosteronism. Front. Endocrinol..

[43] Chen Z., Yang A., Chen A., Dong J., Lin J., Huang C., Zhang J., Liu H., Zeng Z., Miao W. (2024). [^68^Ga]Pentixafor PET/CT for staging and prognostic assessment of newly diagnosed multiple myeloma: Comparison to [^18^F]FDG PET/CT. Eur. J. Nucl. Med. Mol. Imaging.

[44] Ding J., Tong A., Zhang Y., Wen J., Zhang H., Hacker M., Huo L., Li X. (2022). Functional Characterization of Adrenocortical Masses in Nononcologic Patients Using ^68^Ga-Pentixafor. J. Nucl. Med..

[45] Osl T., Schmidt A., Schwaiger M., Schottelius M., Wester H.-J. (2020). A new class of PentixaFor- and PentixaTher-based theranostic agents with enhanced CXCR4-targeting efficiency. Theranostics.

[46] Yang T., Shi D., Lin Q., Shen H., Tan H., Liu Y., Shi H., Cheng D. (2024). Synthesis, Screening, and Evaluation of Theranostic Molecular CPCR4-Based Probe Targeting CXCR4. Mol. Pharm..

[47] Chen C., Ke J., Zhou X.E., Yi W., Brunzelle J.S., Li J., Yong E.-L., Xu H.E., Melcher K. (2013). Structural basis for molecular recognition of folic acid by folate receptors. Nature.

[48] Paulos C.M., Turk M.J., Breur G.J., Low P.S. (2004). Folate receptor-mediated targeting of therapeutic and imaging agents to activated macrophages in rheumatoid arthritis. Adv. Drug Deliv. Rev..

[49] Brand C., Longo V.A., Groaning M., Weber W.A., Reiner T. (2017). Development of a New Folate-Derived Ga-68-Based PET Imaging Agent. Mol. Imaging Biol..

[50] Gnesin S., Müller J., Burger I.A., Meisel A., Siano M., Früh M., Choschzick M., Müller C., Schibli R., Ametamey S.M. (2020). Radiation dosimetry of ^18^F-AzaFol: A first in-human use of a folate receptor PET tracer. EJNMMI Res..

[51] Chen Q., Meng X., McQuade P., Rubins D., Lin S.-A., Zeng Z., Haley H., Miller P., Trotter D.G., Low P.S. (2017). Folate-PEG-NOTA-Al^18^F: A New Folate Based Radiotracer for PET Imaging of Folate Receptor-Positive Tumors. Mol. Pharm..

[52] Clemente G.S., Antunes I.F., Kurhade S., Berg M.P.v.D., Sijbesma J.W., van Waarde A., Buijsman R.C., Willemsen-Seegers N., Gosens R., Meurs H. (2021). Mapping Arginase Expression with ^18^F-Fluorinated Late-Generation Arginase Inhibitors Derived from Quaternary α-Amino Acids. J. Nucl. Med..

[53] Cieslewicz M., Tang J., Yu J.L., Cao H., Zavaljevski M., Motoyama K., Lieber A., Raines E.W., Pun S.H. (2013). Targeted delivery of proapoptotic peptides to tumor-associated macrophages improves survival. Proc. Natl. Acad. Sci. USA.

[54] Huang M., Wang R., Li M., Cai H., Tian R. (2022). Peptide-Based [^68^Ga]Ga Labeled PET Tracer for Tumor Imaging by Targeting Tumor-Associated Macrophages. Pharmaceutics.

[55] Skajaa T., Cormode D.P., Falk E., Mulder W.J., Fisher E.A., Fayad Z.A. (2010). High-density lipoprotein–based contrast agents for multimodal imaging of atherosclerosis. Arter. Thromb. Vasc. Biol..

[56] Pérez-Medina C., Tang J., Abdel-Atti D., Hogstad B., Merad M., Fisher E.A., Fayad Z.A., Lewis J.S., Mulder W.J., Reiner T. (2015). PET Imaging of Tumor-Associated Macrophages with ^89^Zr-Labeled High-Density Lipoprotein Nanoparticles. J. Nucl. Med..

[57] John P., Wei Y., Liu W., Du M., Guan F., Zang X. (2019). The B7x Immune Checkpoint Pathway: From Discovery to Clinical Trial. Trends Pharmacol. Sci..

[58] Kumar M., Singh S.B., Vasyliv I., Habte F., Kalita M., Alam I.S., Koladiya A., Dai S.Y., James M., Rao J. (2025). B7–H4 ImmunoPET Imaging Tracks Tumor-Associated Macrophage Changes in Prostate Cancer. Mol. Pharm..

[59] Gondry O., Xavier C., Raes L., Heemskerk J., Devoogdt N., Everaert H., Breckpot K., Lecocq Q., Decoster L., Fontaine C. (2023). Phase I Study of [^68^Ga]Ga-Anti-CD206-sdAb for PET/CT Assessment of Protumorigenic Macrophage Presence in Solid Tumors (MMR Phase I). J. Nucl. Med..

[60] Gordon S.R., Maute R.L., Dulken B.W., Hutter G., George B.M., McCracken M.N., Gupta R., Tsai J.M., Sinha R., Corey D. (2017). PD-1 expression by tumour-associated macrophages inhibits phagocytosis and tumour immunity. Nature.

[61] Di Conza G., Tsai C.-H., Gallart-Ayala H., Yu Y.-R., Franco F., Zaffalon L., Xie X., Li X., Xiao Z., Raines L.N. (2021). Tumor-induced reshuffling of lipid composition on the endoplasmic reticulum membrane sustains macrophage survival and pro-tumorigenic activity. Nat. Immunol..

[62] Kuang D.-M., Zhao Q., Peng C., Xu J., Zhang J.-P., Wu C., Zheng L. (2009). Activated monocytes in peritumoral stroma of hepatocellular carcinoma foster immune privilege and disease progression through PD-L1. J. Exp. Med..

[63] Binnewies M., Pollack J.L., Rudolph J., Dash S., Abushawish M., Lee T., Jahchan N.S., Canaday P., Lu E., Norng M. (2021). Targeting TREM2 on tumor-associated macrophages enhances immunotherapy. Cell Rep..

[64] Xiang X., Wang J., Lu D., Xu X. (2021). Targeting tumor-associated macrophages to synergize tumor immunotherapy. Signal Transduct. Target. Ther..

[65] Nie R.-C., Hu G.-S., Cao S.-Q., Wang A., Wang D.-C., Liu W. (2026). Spatial single-cell landscape of tumor-associated macrophages and their crosstalk with the tumor microenvironment. Cell Discov..

[66] Ma P., Tan H., Lin Q., Liu Y., Shen H., He S., Su H., Luo Z., Shi D., Cheng D. (2026). Preclinical evaluation of a novel tetrazine probe within a pretargeted delivery system for theranostics in HER2-positive tumor-bearing mice models. Acta Pharm. Sin. B.

[67] Zhang L., Zhou M., Lu C., Zhao P., Shi N., Zhou Y., Yao C., Yang X., Tian J., Wang J. (2025). A high-affinity CEA-targeted nanobody for 68Ga PET imaging and 177Lu-based radioisotope therapy: Preclinical and first-in-human evaluation. J. Nanobiotechnol..

[68] Gómez-Bombarelli R., Wei J.N., Duvenaud D., Hernández-Lobato J.M., Sánchez-Lengeling B., Sheberla D., Aguilera-Iparraguirre J., Hirzel T.D., Adams R.P., Aspuru-Guzik A. (2018). Automatic Chemical Design Using a Data-Driven Continuous Representation of Molecules. ACS Cent. Sci..

[69] Capecchi A., Cai X., Personne H., Köhler T., van Delden C., Reymond J.-L. (2021). Machine learning designs non-hemolytic antimicrobial peptides. Chem. Sci..

[70] Abramson J., Adler J., Dunger J., Evans R., Green T., Pritzel A., Ronneberger O., Willmore L., Ballard A.J., Bambrick J. (2024). Accurate structure prediction of biomolecular interactions with AlphaFold 3. Nature.

[71] Wu Z., Ramsundar B., Feinberg E.N., Gomes J., Geniesse C., Pappu A.S., Leswing K., Pande V. (2017). MoleculeNet: A benchmark for molecular machine learning. Chem. Sci..

